# Abnormal regurgitation in three cows caused by intrathoracic perioesophageal lesions

**DOI:** 10.1186/1751-0147-56-14

**Published:** 2014-03-14

**Authors:** Ueli Braun, Colin Schwarzwald, Stefanie Ohlerth, Sandra Frei, Monika Hilbe

**Affiliations:** 1 Department of Farm Animals, Vetsuisse-Faculty, University of Zurich, Zurich, Switzerland; 2 Clinic of Equine Medicine, Vetsuisse-Faculty, University of Zurich, Zurich, Switzerland; 3 Division of Diagnostic Imaging, Vetsuisse-Faculty, University of Zurich, Zurich, Switzerland; 4 Institute of Veterinary Pathology, Vetsuisse Faculty, University of Zurich, Zurich, Switzerland

**Keywords:** Cattle, Abnormal regurgitation, Oesophagus, Abscess, Tumour

## Abstract

**Background:**

Three Brown Swiss cows with abnormal regurgitation because of a perioesophageal disorder are described.

**Case presentation:**

The cows were ill and had poor appetite, salivation and regurgitation of poorly-chewed feed. Collection of rumen juice was successful in one cow, and in another, the tube could be advanced to the level of the 7th intercostal space, and in the third, only saliva could be collected. In one cow, oesophagoscopy revealed a discoloured 10-cm mucosal area with fibrin deposits. Thoracic radiographs were normal. The cows were euthanased and examined postmortem. Cow 1 had a large perioesophageal abscess containing feed material at the level of the thoracic inlet, believed to be the result of a healed oesophageal injury. Cow 2 had an abscess between the oesophagus and trachea 25 cm caudal to the epiglottis with the same presumed aetiology as in cow 1. Cow 3 had a mediastinal carcinoma that enclosed and constricted the oesophagus.

**Conclusions:**

Abnormal regurgitation in cattle is usually the result of an oesophageal disorder. Causes of oesophageal disorders vary widely and their identification can be difficult.

## Background

Abnormal regurgitation in ruminants is defined as the discharge of food from the mouth [1] and occasionally from the nose [2] immediately after prehension of feed and before the feed enters the forestomachs. Affected cattle may suddenly stop eating, extend the neck momentarily and after a brief episode of retching, eject partially chewed food mixed with saliva. Regurgitation must be differentiated from vomiting; the latter follows a short period of restlessness and is characterised by a considerable amount of forestomach ingesta exiting the mouth or nose with force [1]. The vomitus of ruminants contains rumen fluid and small feed particles. While regurgitation is a normal phenomenon in ruminants, abnormal regurgitation is usually the result of an oesophageal disorder, often affecting the intrathoracic part. Causes include oesophageal dilatation [3-7] and narrowing [8], various types of trauma, chemical irritation, infection and parasites [1]. Causes of vomiting are more numerous and have been summarized in detail [1]. Briefly, vomiting may be centrally mediated involving the stimulation of chemoreceptors in the brain stem or it may occur in response to stimulation of peripheral receptors in the digestive tract. Overall, regurgitation and vomiting are uncommon in cattle, and a PubMed literature search of the last 20 years using the MeSH regurgitation, regurgit*, vomiting, vomit*, cow*, cattle* yielded only one publication of a cow with vomiting attributable to abomasal lymphosarcoma [9]. This report describes three cows in which the main clinical sign was abnormal regurgitation due to oesophageal disorders with different aetiologies.

### History and clinical findings

Three Brown Swiss cows, which originated from dairy herds with a mean milk yield of 7’800 to 8’500 kg per lactation and ranged in age from six years (cow 1) to nine years (cows 2 and 3), were referred to our clinic because of abnormal regurgitation that had not responded to treatment. Cow 1 was eight months pregnant and had reduced appetite and abnormal regurgitation for one week before admission. The referring veterinarian was able to pass a small-bore stomach tube into the rumen without difficulty but not a large-bore tube. The cow was treated with a nonsteroidal anti-inflammatory drug. Cow 2 was two months fresh and had reduced appetite, excessive salivation and abnormal regurgitation, which started two days before referral. The cow was treated with a magnet and a spasmolytic drug. Cow 3 was one week fresh and had abnormal regurgitation and salivation, which started several weeks before calving. The cow was treated with an antibiotic for several days, but there was no improvement.

All cows had an abnormal general condition and demeanour, were listless and had moderate (cows 1 and 2) to severe (cow 3) reduction in feed intake. The rectal temperature (38.7, 38.4, 38.4°C), heart rate (72, 68, 60 beats/min) and respiratory rate (24 breaths/min) were normal. Cows 2 and 3 had reduced ruminal contractions, mild ruminal tympany, sunken eyes and reduced skin turgor. Cow 1 had no ruminal contractions and bruxism. Cow 2 had droopy ears and severely injected scleral vessels. Furthermore, cow 2 had ructus-like peristalsis of the oesophagus and pharynx every 15 to 20 sec during examination. Transrectal examination revealed an L-shaped, tympanic rumen and thin to watery faeces. Cow 3 had a cold muzzle and uraemic breath.

**Figure 1:**
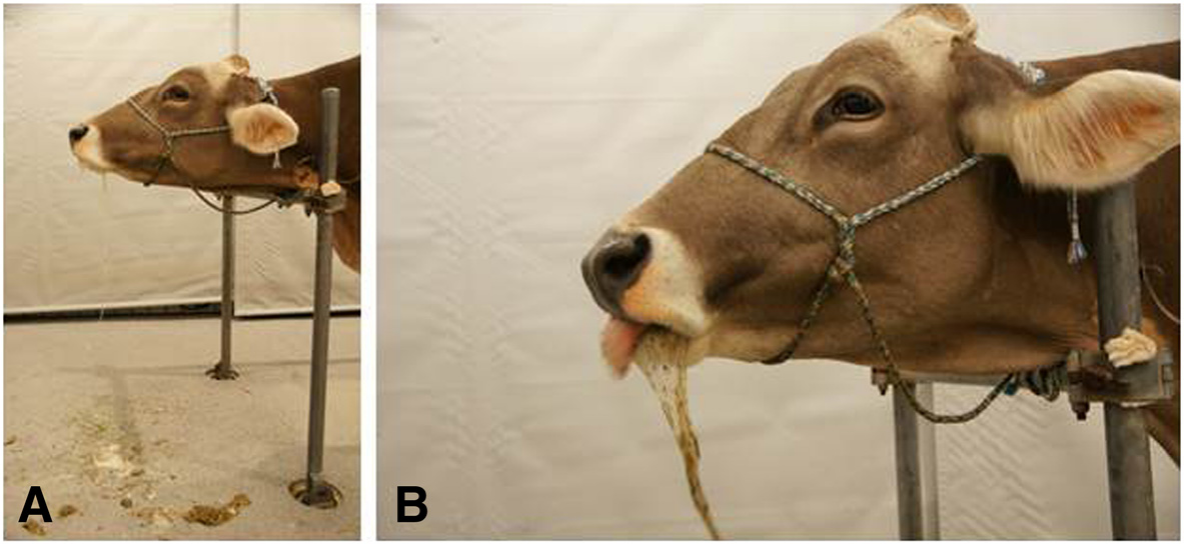
**Abnormal regurgitation in a cow with perioesophageal abscess.** Abnormal regurgitation in a 6-year-old Brown Swiss cow (cow 1) with perioesophageal abscess. **A)** The head and neck of the cow are extended; the floor is littered with regurgitated partly-chewed feed and saliva, **B)** Close-up: Head and neck are extended; regurgitation of saliva and feed.

Cow 1 ate hay readily but regurgitated immediately after the first mouthful, which included retching, salivation and dropping feed from the mouth over a period of about 30 minutes (Figure 1A, B). A video of the case is shown in Additional file 1. These signs were accompanied by repeated coughing and vocalisation. Foamy saliva and pieces of partly chewed feed accumulated in the manger overnight.

Cow 2 was observed over a period of ten days, during which time the feed intake was about 50% of normal. There was recurrent, mild, self-limiting, ruminal tympany. There were several episodes of sudden abnormal regurgitation of feed immediately after swallowing, which were followed by episodes of apparently normal eating behaviour.

The appetite of cow 3 was markedly reduced, and thus regurgitated material consisted mostly of saliva with very little feed.

The clinical examination of the cranial nerves of all cows yielded no further abnormalities and there were no ataxia and any other signs of central nervous disorders. Listeriosis was excluded in all cows.

### Examination of blood and rumen fluid

Abnormal haematological findings included mild anaemia (cow 1), increased aspartate-aminotransferase activity (cow 3) and hypokalaemia (cow 3) (Table 1). Mild leukopenia (cows 1, 3), hyperproteinaemia (cows 2, 3), hyperfibrinogenaemia (cows 2, 3), mild azotaemia (cows 1, 3) and hypocalcaemia (cows 1, 3) were additional findings. Sodium and inorganic phosphorus concentrations were lower than normal in all cows, and cows 2 and 3 also had mild metabolic acidosis (Table 2). Cow 1 was the only cow in which rumen juice could be collected using a stomach tube; the fluid was olive green, had a stale smell, was inactive and had a high-normal chloride concentration of 30 mmol/l. In cow 2, three attempts were made to pass a stomach tube, but it could not be advanced beyond the level of the 7th intercostal space. Only saliva could be collected from cow 3.

**Table 1:** Haematological and biochemical results in 3 cows with perioesophageal disorders

**Variable**	**Cow 1**	**Cow 2**	**Cow 3**	**Normal range**
Haematocrit (%)	29	33	35	30 – 35
Total leukocyte count (× 10^3^/μl)	4.3	8.6	4.3	5.0 – 10.0
Total protein (g/l)	70	96	90	60 – 80
Fibrinogen (g/l)	2	6	10	4 – 7
Urea (mmol/l)	6.9	4.0	8.3	2.4 – 6.5
ASAT (U/l)	40	86	256	20 – 103
γ-GT (U/l)	25	25	25	9 – 30
Sodium (mmol/l)	144	142	144	145 – 155
Potassium (mmol/l)	4.0	4.2	3.5	4 – 5
Chloride (mmol/l)	104	104	106	96 – 105
Calcium (mmol/l)	2.24	2.59	2.14	2.3 – 2.6
Inorg. phosphorus (mmol/l)	1.25	0.83	1.16	1.3 – 2.4
Magnesium (mmol/l)	0.85	0.92	0.96	0.8 – 1.0
Rumen chloride (mmol/l)	30	CNBC	CNBC	15 – 30

CNBC Could not be collected.

**Table 2:** Venous blood gas analysis in 3 cows with perioesophageal disorders

**Variable**	**Cow 1**	**Cow 2**	**Cow 3**	**Normal range**
pH	7.42	7.40	7.33	(7.41 – 7.45)
pCO_2_ (mmHg)	38	32.4	36.9	(35 – 45)
Bicarbonate (mmol/l)	23.7	20.9	19.8	(20 – 30)
Base excess (mmol/l)	0.2	– 3.8	– 6.0	– 2 to +2

### Endoscopic, ultrasonographic and radiographic examination

**Figure 2:**
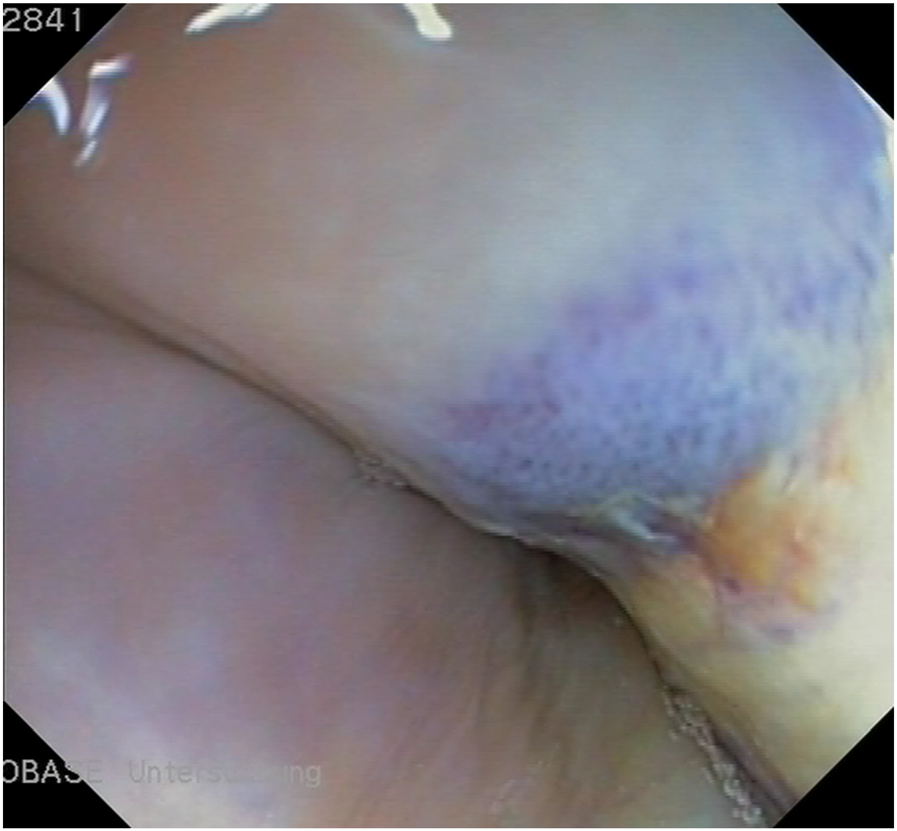
**Oesophagoscopy in a Brown Swiss cow with a perioesophageal abscess.** Oesophagoscopy in a 6-year-old Brown Swiss cow (cow 1): There is a 10-cm area of dark red to blue mucosal discolouration and fibrinous deposits 110 cm from the muzzle.

Endoscopic examination (video coloscope, 1680 mm × 11.5 mm, Olympus Schweiz, PCF-Q-180AL) of the trachea of all cows revealed no abnormal findings, and examination of the oesophagus was also normal in cows 2 and 3. In cow 1, the entire oesophagus was patent, but there was a 10-cm area of dark red to blue mucosal discolouration and fibrinous deposits 110 cm from the muzzle (Figure 2). Megaoesophagus could be ruled out in all cows.

The reticulum could not be viewed ultrasonographically in cow 1. In cow 2, the reticular contour appeared normal and there were 4 biphasic reticular contractions in 3 min (normal, 3 to 4 contractions/3 min) [10]. The reticulum of cow 3 was elevated from the ventral abdominal wall and had no contractions. There was a small amount of anechoic fluid and echoic fibrin caudal to the reticulum.

Radiographs of the lungs and reticulum were normal in cows 1 and 2. Only the reticulum was examined in cow 3 and it was elevated from the ventral abdominal wall and contained a magnet.

The clinical examination of the cranial nerves of all cows yielded no further abnormalities and there were no ataxia and any other signs of central nervous disorders.

### Clinical diagnosis, treatment and postmortem examination

A clinical diagnosis of abnormal regurgitation was made in all three cows, and based on information from standard texts [1,2] an association with oesophageal disease was assumed even though only cow 1 had endoscopic evidence of this. This cow received 10 l of a NaCl-glucose solution (50 g glucose and 9 g sodium chloride/l), administered slowly via an indwelling intravenous catheter daily for three days, amoxicillin (7 mg/kg, Clamoxyl®, Zoetis Schweiz, Zürich) administered intramuscularly for 3 days, and 500 ml of 40% calcium borogluconate (Calcamyl-40MP®, Graeub, Bern) administered once intravenously. However, she did not respond to treatment. Cow 2 was also treated with the same NaCl-glucose solution, 500 mg flunixin meglumine (Fluniximin®, Graeub, Bern) intravenously for three days, and amoxicillin intramuscularly for 10 days, after which she was discharged at the owner’s request. The cow was referred to our clinic four weeks later for euthanasia because of deteriorating condition and severe weight loss. Cow 3 received the same treatment as cow 2, and because there was no response, was euthanased. All three cows underwent postmortem examination.

**Figure 3:**
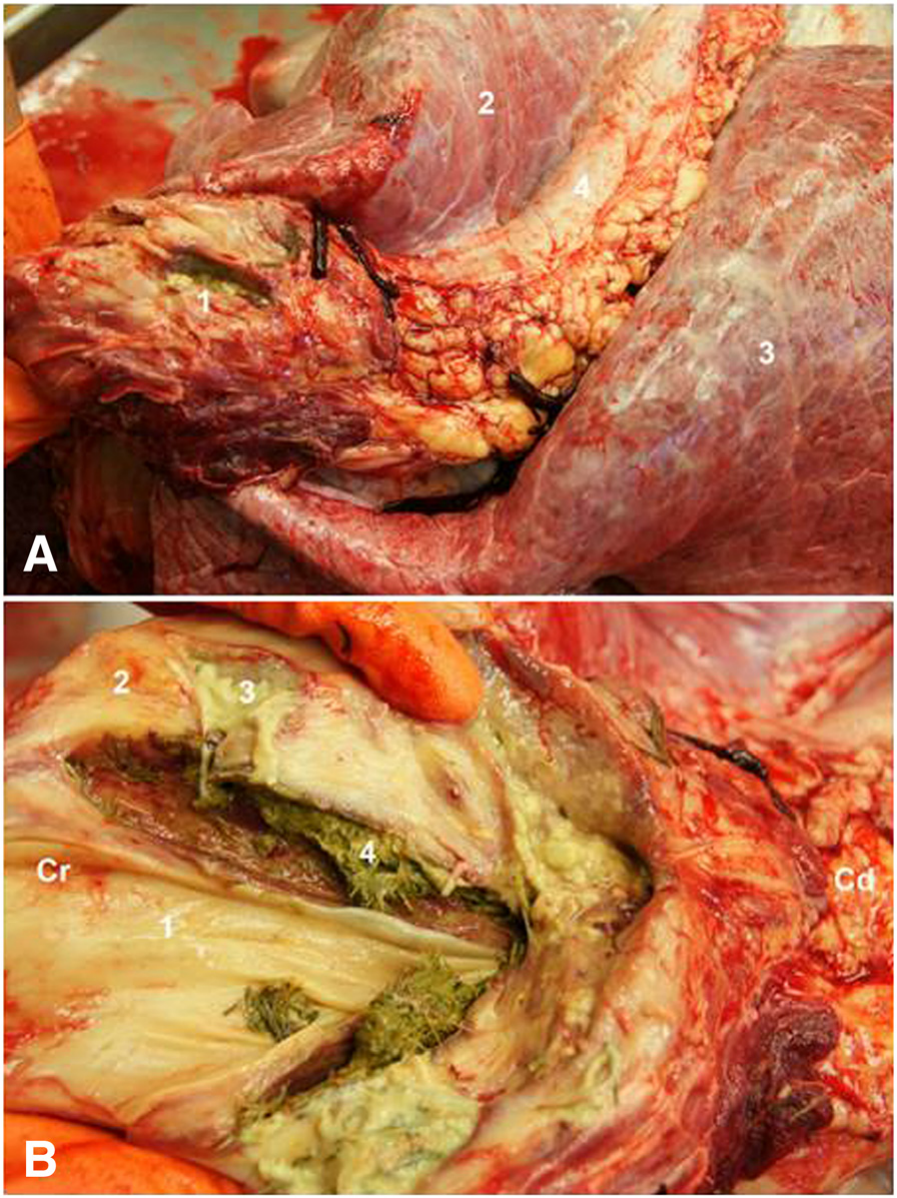
**Perioesophageal abscess in a Brown Swiss cow.** Perioesophageal abscess in a 6-year-old Brown Swiss cow (cow 1). **A)** Abscess measuring 10 cm by 15 cm in the cranial mediastinum (1). Other structures in the figure are left lung lobe (2), right lung lobe (3), trachea (4). **B)** Close-up of the specimen shown in A: The oesophagus (1) and the abscess (2) have been opened; the abscess contains pus (3) and feed (4).

**Figure 4:**
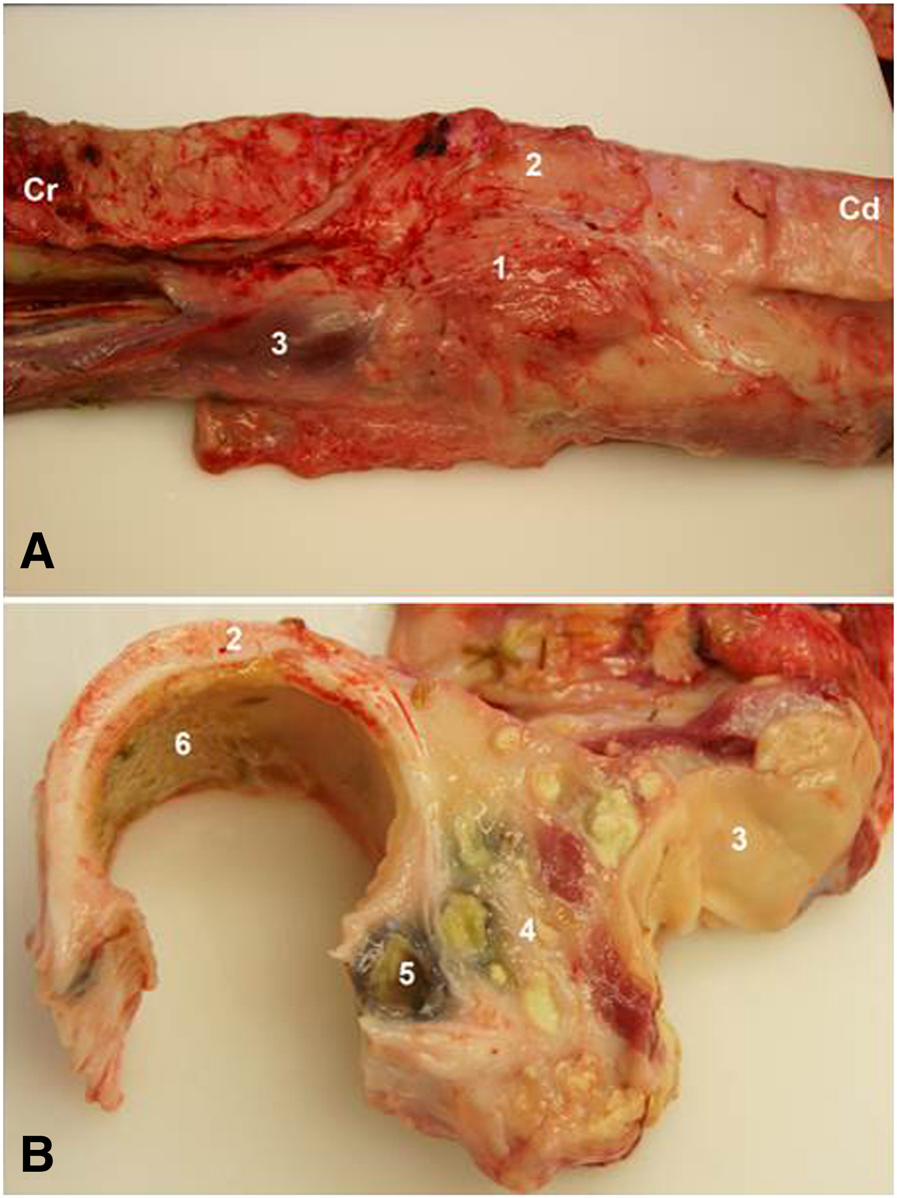
**Abscess between the trachea and oesophagus in a Brown Swiss cow. A)** An abscess (1) is seen between the trachea (2) and oesophagus (3) in a 9-year-old Brown Swiss cow (cow 2), Cr: cranial, Cd: caudal. **B)** Cross section of the abscess showing multiple pus-filled cavities (4), an abscess that had ruptured into the trachea (5) and purulent material on the mucosa (6).

**Figure 5:**
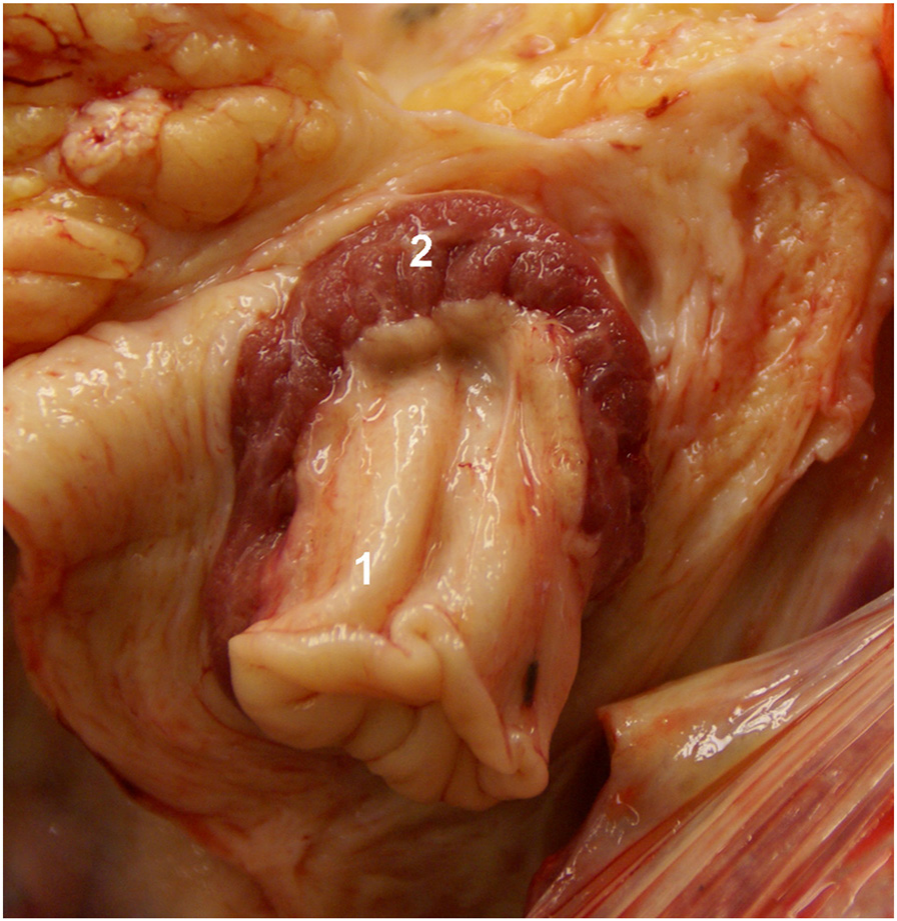
**Carcinoma enclosing and constricting the oesophagus in a Brown Swiss cow.** Oesophagus (1) enclosed and constricted by carcinoma (2) showing infiltrative growth in the tunica muscularis in a 9-year-old Brown Swiss cow.

**Figure 6:**
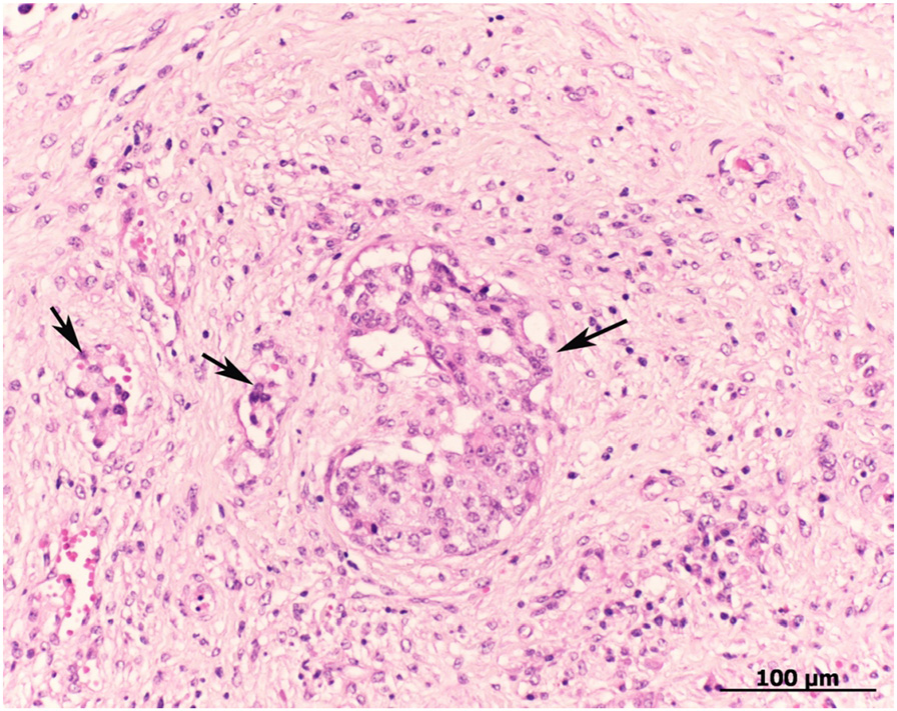
**Histological findings in a Brown Swiss cow with scirrous carcinoma enclosing and constricting the oesophagus.** Scirrhous carcinoma consisting of small groups of mostly solid strands of epithelial cells (arrows) surrounded by abundant connective tissue, lymphocytes, plasma cells and few blood vessels. Haematoxylin and eosin.

In cow 1, massive fibrinous adhesions were seen between the oesophagus and surrounding tissue as well as the trachea and surrounding tissue. This area also had a large well-encapsulated abscess (Figure 3A) containing feed material (Figure 3B). The oesophageal mucosa was macroscopically normal. A postmortem diagnosis of perioesophageal abscess, presumably as a result of oesophageal trauma, was made. Other findings included adhesions between the diaphragm and liver and between the diaphragm and intestines, and two non-perforating abomasal ulcers measuring 5 mm and 10 mm in diameter. Cow 2 had an abscess between the oesophagus and trachea, 25 cm caudal to the epiglottis (Figure 4A, B). The abscess was 8 cm by 15 cm and consisted of multiple cavities, which were 1 cm to 3 cm in diameter and contained white creamy to yellowish-green purulent material. The abscess also involved the oesophageal wall and had ruptured into the trachea. The tracheal wall next to the abscess was thickened and the mucosa was covered with pus (Figure 4B). The postmortem diagnosis was abscess between the oesophagus and trachea, presumably as a result of oesophageal trauma. There was also severe purulent bronchopneumonia. Cow 3 had a mediastinal mass that enclosed and constricted the oesophagus (Figure 5). The mass was reddish brown and firm and had a greasy appearance on cut surface. There were similar lesions in the diaphragm and greater omentum. Histological examination of these masses revealed multifocal accumulations of slightly polymorphic, polygonal cells containing a moderate amount of eosinophilic cytoplasm and a nucleus that was often centrally located with a distinct nucleolus of varying size. Mitotic figures were scant. The epithelial cells formed solid strands or occasionally tubular structures and were seen in lymphatic vessels in some places. The tumour cells were surrounded by abundant fibrovascular stroma, numerous lymphocytes and occasional plasma cells. The histological diagnosis was scirrhous carcinoma (Figure 6).

## Discussion

The clinical diagnosis of abnormal regurgitation was straightforward in all three cows, but the frequency and severity of clinical signs varied considerably among the patients. Cow 1 regurgitated after every mouthful of food and cow 2 only intermittently. The third cow was almost anorectic and regurgitation was episodic. This cow originated from a free stall in which regurgitated feed material had been noticed for several weeks, but it took the owner some time to identify its source. Differentiation of vomiting and regurgitation based on clinical signs was also straightforward because of the close temporal relationship between the prehension of feed and ejection, and because the feed was poorly chewed and contained saliva. Vomitus of ruminants contains finely chewed feed particles and rumen juice [1]. The differential diagnosis of regurgitation of partly chewed feed includes jaw fractures, dental disease, listeriosis, botulism, brain stem abscess and other disorders that lead to quidding. These disorders were ruled out by observing the patients during eating.

The cows underwent considerable diagnostic workup, but most findings were of limited usefulness. The haematological findings did not aid in identifying the cause of regurgitation. There were a few mild abnormalities including hypophosphataemia in all three cows, which was attributable to salivary loss of phosphorus [11]; salivary phosphorus is normally resorbed in the rumen and small intestine. Likewise, the loss of bicarbonate in the saliva accounted for the mild metabolic acidosis. Because the cause of abnormal regurgitation is usually found in the oesophagus [1,2], particularly the intrathoracic section [1], the purpose of further diagnostic procedures was to identify intrathoracic lesions associated with the oesophagus. A simple procedure was to pass stomach tubes of different diameters. This was informative in cow 1, in which a small-bore tube, but not a large-bore tube, could be passed into the rumen, and also in cow 2, in which several attempts consistently indicated a blockage at the level of the 7th intercostal space. Although oesophagoscopy has been an established diagnostic technique in cattle for several years [12], the information gained from endoscopy was disappointing. An oesophageal constriction was not detected in any of the cows; the only lesion detected was discolouration of the mucosa and fibrinous deposits at the level of the abscess, pointing to a healed injury in cow 1. It was understandable that the oesophageal narrowing caused by a perioesophageal abscess in cows 1 and 2 could not be seen endoscopically, because the abscesses were located perioesophageally and did not surround the entire oesophagus; however, it was surprising that no constriction was seen in cow 3, in which a neoplasm surrounded the oesophagus in a collar-like fashion. Likewise, no abnormalities suggesting a mass or gas from an abscess were seen on thoracic radiographs. It is possible that oesophageal narrowing could have been detected using a barium study. It was not surprising that a perioesophageal lesion was the cause of abnormal regurgitation in all three patients, and this finding was in agreement with standard veterinary texts [1,2]. Vomiting on the other hand has a variety of causes [1]. The lesion responsible for the oesophageal narrowing was thought to have originated from oesophageal trauma in cows 1 and 2 and was neoplastic in cow 3.

## Conclusions

Abnormal regurgitation in cattle is usually the result of an oesophageal disorder. Causes of oesophageal disorders vary widely and their identification can be difficult. Because techniques such as endoscopy and radiography may not yield diagnostic information, careful clinical examination including probing the oesophagus with a stomach tube is critical.

## Competing interests

The authors declare that they have no competing interests.

## Authors’ contributions

UB was responsible veterinarian for the patients and the ultrasonography and drafted the manuscript, CS was responsible for the endoscopy and SO was responsible for the radiographic examination. SF was involved in the clinical examination of the cows while MH did the postmortem examinations. All authors have read and approved the final version of the manuscript.

## Supplementary Material

Additional file 1 **Abnormal regurgitation in a Brown Swiss cow with perioesophageal abscess.** Abnormal regurgitation in a 6-year-old Brown Swiss cow (cow 1) with perioesophageal abscess. The cow readily eats hay. Immediately afterwards, the cow becomes anxious and distressed and retches, regurgitates, coughs and groans. Click here for file
